# Mindfulness based cognitive therapy for recurrent depressive disorder

**DOI:** 10.1192/bjo.2021.724

**Published:** 2021-06-18

**Authors:** James McLoughlin, Paula Martin, Geraldine McCarthy, Chee Lin Piong

**Affiliations:** 1 University Hospital Galway; 2 Beo Mindful Health; 3 Sligo University Hospital

## Abstract

**Aims:**

Mindfulness-based therapies have been demonstrated to be effective in reducing anxiety, stress and depressive symptoms in adults. Depression is a chronic relapsing condition. Major depressive disorder is one of the most common causes of ill health and functional impairment.

Our goal was to assess the real world clinical effectiveness of Mindfulness Based Cognitive Therapy (MBCT) for Recurrent Depressive Disorder in three domains:

-Depression, anxiety and stress levels

-Mindfulness level

-Self-compassion level

**Method:**

Patients with a diagnosis of Recurrent Depressive Disorder (primary or secondary diagnosis) were referred by their community mental health team to participate in an 8-week educational MBCT programme. Participants completed the Depression, Anxiety and Stress (DASS), 5-Facet Mindfulness and Self Compassion self-rated scales prior to commencing and at the end of the course. They were also invited to give qualitative feedback at the end of the course.

Data were collected from four groups who completed the course over a period of twelve months. A paired samples test was used to compare pre and post intervention scores to determine effect size.

**Result:**

We had complete data for 19 participants out of a cohort of 34. Pre intervention scores were similar for both groups.

The mean age of the cohort was 47 years (SD of 10 years), 3 male, 16 female.

Patients showed a clinically significant reduction of symptoms in depression, anxiety and stress, with respective reductions of 48%, 26% and 43% post intervention. Results were statistically significant for depressaion and stress p <0.001 but not for anxiety p = 0.130.

Positive trends were seen in all domains of the 5-Fact Mindfulness and Self Compassions scales, with mean improvements of 28.2% and 35.3% respectively. All results were statistically significant.

We also collected anonymized qualitative feedback which highlighted themes of empowerment, skill acquisition and improved coping.

**Conclusion:**

Numerous studies have demonstrated poor compliance with antidepressant treatments commonly prescribed in Recurrent Depressive Disorder. This small scale study demonstrates a statistical and clinical benefit of MBCT for these patients, supporting greater use of such novel non-pharmacological therapeutic options as treatment strategies..

